# Cross Cultural Adaptation and Validation of Italian Version of the Leeds Assessment of Neuropathic Symptoms and Signs Scale and Pain DETECT Questionnaire for the Distinction between Nociceptive and Neuropathic Pain

**DOI:** 10.1155/2021/6623651

**Published:** 2021-04-28

**Authors:** Alberto Migliore, Gianfranco Gigliucci, Antimo Moretti, Alessio Pietrella, Marco Peresson, Fabiola Atzeni, Piercarlo Sarzi-Puttini, Laura Bazzichi, Sara Liguori, Giovanni Iolascon

**Affiliations:** ^1^Rheumatology Unit, San Pietro Fatebenefratelli Hospital, Rome, Italy; ^2^Department of Medical and Surgical Specialties and Dentistry, University of Campania “Luigi Vanvitelli”, Naples, Italy; ^3^Neurological Section, San Pietro Fatebenefratelli Hospital, Rome, Italy; ^4^Rheumatology Unit, University of Messina, Messina, Italy; ^5^Rheumatology Unit, ASST-Fatebenefratelli-L. Sacco University Hospital, Milan, Italy; ^6^Rheumatology Unit, Department of Clinical and Experimental Medicine, University of Pisa, Pisa, Italy

## Abstract

**Objective:**

This study aimed to validate Italian versions of Leeds Assessment of Neuropathic Symptoms and Signs (LANSS) scale and Pain DETECT questionnaire (PD-Q) and evaluate the ability of these questionnaires to discriminate between nociceptive and neuropathic pain.

**Design:**

Multicenter prospective validation cohort study. *Subjects and Setting.* One hundred patients were included with a diagnosis formulated by a specialist in outpatient settings (50 affected by knee osteoarthritis as nociceptive pain and 50 affected by trigeminal or postherpetic neuralgia as neuropathic pain).

**Methods:**

The Italian versions of both questionnaires according to Italian cultural characteristics were performed according to the following steps: (1) translation of the questionnaires from English into Italian; (2) review by a bilingual individual for consistency; (3) proposed version after a mail round between experts; (4) backward translation; (5) comparison with the original English version by the experts; (6) approved version of the questionnaires. One hundred patients were enrolled and completed the two questionnaires administered by a specialist or blinded nursing staff, at the baseline and after 24/48 hours. Internal consistency, stability, validity, and discriminative power were analyzed.

**Results:**

Statistically significant differences were reported about the ability of both questionnaires to discriminate between patients affected by neuropathic or nociceptive pain. Internal consistency for the Italian version of the LANSS was 0.76, and for PD-Q, it was 0.80, assessed by Cronbach's *α*; LANSS showed a good test-retest reliability with an ICC of 0.76, and PD-Q showed a high test-retest reliability with an ICC of 0.96. For interrater reliability, there was a concordance rate of 83.3% between reference diagnosis and LANSS (Cohen's kappa = 0.67, CI 95% 0.52–0.75).

**Conclusions:**

This study validated the Italian versions of LANSS and PD-Q as reliable instruments with good psychometric characteristics, for pain evaluation, discriminating between nociceptive and neuropathic pain. Our findings were similar to those observed in the original study. Furthermore, we have reported the test-retest reliability for both questionnaires, not addressed in original validation studies.

## 1. Introduction

Pain treatment is a key element in the management of many chronic diseases. In this regard, traditional therapeutic strategies addressed pain intensity, while, recently, a more appropriate approach taking into account the pathogenic mechanisms of pain has been widely used [1]. However, in clinical practice, many analgesic drugs are still prescribed without adopting pathogenic criteria with a consequent increased risk of subtherapeutic responses [2].

This is especially true when it comes to treating neuropathic or mixed pain. To correctly identify patients who can respond to a specific intervention, validated assessment tools have been produced. In particular, several questionnaires have been created and validated in different languages to discriminate between different types of pain, such as the Pain DETECT questionnaire (PD-Q) and the Leeds Assessment of Neuropathic Symptoms and Signs (LANSS) scale [3, 4], which can be used in the challenging diagnosis of neuropathic pain (NeP). Nowadays, an Italian version of these questionnaires is not available; their translations and cross-cultural adaptations might be useful for Italian physicians to improve the management of different types of pain in clinical practice.

The objectives of this study were to translate and validate Italian versions of the LANSS and PD-Q scales, as well as to investigate the ability of these tools to discriminate between inflammatory/mechanical (nociceptive) and NeP.

## 2. Methods

A multicenter prospective cohort study was conducted with an overall duration of 8 months (4 months of observation, 3 months of enrollment, and 1 month of data compilation), involving 5 outpatient services belonging to the Italian Society for Unified and Interdisciplinary Management of Musculoskeletal Pain and Algodystrophy (Società Italiana per la Gestione Unificata e Interdisciplinare del Dolore muscolo-scheletrico e dell'Algodistrofia, SI-GUIDA) for the inclusion of 100 patients.

Italian versions of both questionnaires were first developed through translation and back-translation and then analyzed for internal consistency, stability, validity, and discriminative power.

The guidelines of Beaton et al. [5] were used for validation, applying a translation and cultural adaptation. A first translation of the questionnaires was performed from the original English versions according to Italian cultural characteristics by three medical translators, reviewed by a bilingual individual to evaluate conceptual errors or inconsistencies. The proposed version of the questionnaires was revised in a mail round between experts involved in this study. Then, two English native speakers, with no access to the original version, performed backward translations. New versions were compared to the original ones by an expert involved in the study, and then, approved version of the questionnaires was produced. Finally, the definitive Italian version was validated in a clinical setting.

In this study, we considered three paradigmatic diseases causing nociceptive or neuropathic pain, namely, osteoarthritis, and trigeminal or postherpetic neuralgia, respectively.

A series of 100 subjects, of which 50 with nociceptive pain and 50 with NeP were included. Sociodemographic information on the patient and data about the general characteristics of the index disease was collected (date of diagnosis and of symptoms onset, and clinical significance). The patients enrolled in the validation study were selected by the specialist of the different centers in the routine clinical practice. Inclusion criteria were age ≥18 years; diagnosis of trigeminal neuralgia or postherpetic neuralgia (NeP group) or a diagnosis of knee osteoarthritis (OA) according to American College of Rheumatology (ACR) criteria [6] (nociceptive pain group), for at least 3 months; pain intensity assessed according to Visual Analogue Scale (VAS) at baseline ≥40 mm and persistence of symptoms for at least 3 months; complete ability to understand and speak the Italian language; patients able to adhere to the study procedures; patients able to understand and sign informed consent. Exclusion criteria were adults with mixed features of pain; cancer pain or fibromyalgia; presence of other joint diseases (rheumatoid arthritis; spondyloarthritis; connectivitis; polymyalgia rheumatica; gout; Paget's disease of bone; history of septic arthritis; fractures; osteonecrosis); patients taking analgesics or intra-articular treatments; impaired cognitive status; any sensory impairment that may interfere with the compilation of the questionnaire (blindness, deafness); poor or inadequate ability to understand and speak Italian language.

The diagnosis formulated by the specialist was considered as the “reference diagnosis” for the validation of the questionnaires. At the end of the specialist visit, the patient was asked to participate in the study. In case of acceptance, the patient signed an informed consent in order to be included in the study and to allow the use of the collected data. The study protocol and informed consent forms of the study were submitted to and approved by the local ethics committee, in accordance with the ethical principles originating from the Declaration of Helsinki. The questionnaires were administered to patients selected by another specialist or blinded nursing staff. Each patient filled out the questionnaires twice, at baseline and after 24–48 hours. In addition, the personnel who administered the questionnaires had to answer a series of questions about the time needed to complete the questionnaire, the patient's ability to answer without help, and the grade of difficulty in understanding each item. A note was made to report difficulties encountered by each patient. The results of each questionnaire were calculated according to the original scoring system and assessed taking into account the “reference diagnosis” formulated during the baseline visit.

To minimize the risk of short-term clinical change, treatment of these patients remained constant during the study period.

### 2.1. Outcome Measures

#### 2.1.1. LANSS Scale

The LANSS scale is a brief and easily applied instrument including the assessment of 5 symptoms and 2 signs. It is a semistructured interview, in which the patient is asked whether the description presented matches the pattern of pain felt during the past week. The original version was tested and validated in several settings with sensitivity ranging between 82% and 91% and specificity ranging from 80% to 94% compared with clinical diagnosis [4].

The LANSS includes a total of 7 items grouped in 2 sections. The first section (Section A) consists of 5 questions scored from 1 to 5 depending on the items whenever the answer is affirmative, and 0 if this is absent. The following descriptors are most frequently used by patients with NeP: bursting, electric shocks, changes in skin temperature/color, and others. It is the only neuropathic screening that investigates autonomic changes [7]. Section B refers to the physical examination, in which the sensorial characteristics of pain, such as allodynia and hyperalgesia, are explored by means of skin stimulation (stroking cotton wool and pinprick): score is 0 if the answer is negative, and 5 or 3, depending on the question, if positive. Therefore, the sum of all points obtained on the different items of both sections may vary between 0 and 24, and a cutoff point of 12 has been set as being indicative of NeP.

#### 2.1.2. PD-Q

The PD-Q was originally developed for people with low-back pain and showed good sensitivity (85%) and specificity (80%) when compared to clinical diagnosis of a predominantly nociceptive (e.g., visceral-pain) or neuropathic (e.g., postherpetic neuralgia) origin [3]. The PD-Q classifies people into different groups according to a summative score from nine-items: NeP component is unlikely (≤12), result is ambiguous [8–13], and NeP component is likely (≥19). Most items use a 6-point scale, where higher scores are suggestive of greater intensity.

### 2.2. Statistical Analysis

Statistical analyses were performed using SPSS version 15.0 for Windows (Statistical Package for Social Sciences. Inc., Chicago, IL, USA). All continuous data were described by mean ± standard deviation and qualitative data by percentage. The significance of the differences between continuous variables was analyzed using the one-way analysis of variance (ANOVA) and Student's *t*-test (two independent sample *t*-tests were used), while categorical variables were analyzed with chi-squared test.

For reliability analysis, Cronbach's *α* was used to assess the internal consistency [14], and intraclass correlation coefficient (ICC) between test and retest scores was used to assess stability over time [15]. The correlation between the assessment tools (PD-Q and LANSS) and clinical diagnosis was calculated using Pearson's correlation coefficient, and diagnostic classification of patients according to questionnaires was compared with clinical judgment.

In all analyses, *p* values <0.05 were considered statistically significant.

## 3. Results

The study involved 100 patients, 50 patients affected by NeP (trigeminal or postherpetic neuralgia) (15 men and 35 women, mean age 50 years, range 21–85 years) and 50 patients affected by nociceptive pain (knee OA) (20 men and 30 women, mean age 60 years, range 50–70 years) (see Table 1 for further details).

The Italian translations of the questionnaires corresponded extremely well to the original versions, and no problems were reported by patients in the compilation. Questions results were clear and relevant for the description of pain.

Analyzing the psychometric properties of both questionnaires, all items of LANSS scale showed a strong factor loading with the first factor (ranging from 0.65 to 0.96). Cronbach's alpha value was 0.76 for the total LANSS score. Regarding LANSS stability, 100 patients have been retested within 48 hours, and the scores have shown high stability (ICC = 0.76). For the validity of the LANSS scale, using a cutoff ≥12, we obtained a sensitivity of 87% and specificity of 72%; the overall classification was 82%, positive predictive value (PPV) was 83%, and negative predictive value (NPV) was 79%. The diagnostic concordance rate between the clinical diagnosis and the LANSS scale was 83.3% (Cohen's kappa = 0.67, CI 95% 0.52–0.75).

For the validity of PD-Q, we obtained a sensitivity of 85% and specificity of 75%; the overall classification was 81%, positive predictive value (PPV) was 82%, and negative predictive value (NPV) was 76%. The diagnostic concordance rate between the clinical diagnosis and the PD-Q was 83.5% (Cohen's kappa = 0.68, CI 95% 0.54–0.75).

The internal consistency of the Italian version of PD-Q was 0.80 assessed by Cronbach's *α*. The mean overall score of the PD-Q was 16.33 ± 8.35 at baseline and 15.9 ± 8.17 when performing the retest measurement. The PD-Q test-retest reliability had a result of 0.96 for the total score, ranging from 0.84–0.96 for individual items. No floor or ceiling effect was observed.

Statistically significant differences were reported about the capability of the questionnaires to differentiate patients affected by neuropathic or nociceptive pain, without differences in the same type of pain between the results reported at baseline and at retest (Table 2). Specifically, as reported in Table 2, according to pain reference diagnosis, NeP group showed a mean PD-Q score of 22.35 ± 4.8 (retest 21.5 ± 4.1), while NoP group reported a mean score of 4.8 ± 3.7 (retest 5.2 ± 3.5), with a cutoff suggestive of NeP of ≥19. Regarding the LANSS score, NeP group showed a mean score of 18 ± 4.8 (retest 17.5 ± 4.6), while NoP group reported a mean value of 4.4 ± 3.4 (retest 4.5 ± 3.7), with a cutoff point of 12 as indicative of NeP.  Table 3 shows the results of PD-Q in the NeP and the nociceptive pain group, respectively.

## 4. Discussion

This study was designed to validate the Italian versions of LANSS and PD-Q by analyzing the psychometric properties of these questionnaires and to define the ability of these diagnostic tools in discriminating between nociceptive and NeP.

Our results demonstrated a correspondence of Italian translations of LANSS and PD-Q to the original versions; these questionnaires were well accepted by the patient even when readministered after 24/48 hours, with a good patients' reading comprehension and ability to answer.

In particular, the good psychometric properties of the original version have been confirmed for both questionnaires, with a consistent capacity to distinguish between nociceptive and NeP. Moreover, both LANSS and PD-Q were shown to be extremely stable at test-retest analysis, a psychometric property not assessed in the original reference studies [3, 4].

This study confirmed the validity and reliability of these questionnaires for comprehensive NeP assessment.

For reliability, LANSS internal consistency was determined as acceptable with a Cronbach's-*α* of 0.76 in line to the result demonstrated in the original study (Cronbach's-*α* of 0.74) [4], while a lower Cronbach's-*α* value (0.65) was shown by Batistaki et al. in the Greek LANSS validation study [16].

For PD-Q, there was a good internal consistency with Cronbach's-*α* value of 0.80, and the observed value was similar to that observed in the original study (7 Likert items, Cronbach's-*α* value: 0.83) [3] and in the Spanish PD-Q version (whole scale Cronbach's-*α* value of 0.86; 7 Likert items, Cronbach's-*α* value of 0.89, respectively) [17]. Two questionnaires confirmed the strong ability to distinguish between nociceptive and NeP, and to identify the neuropathic component in painful conditions with similar results showed at the retest.

Our results demonstrated a good test-retest reliability for LANSS (ICC 0.76). The original LANSS version did not assess test-retest reliability [4], and our findings are similar to those of Batistaki et al. that showed a strong correlation between the two evaluations (*r* = 0.94, *p* < 0.001) in Greek translation of LANSS [16].

Similarly, for PD-Q, we found a high test-retest reliability (ICC 0.96, range 0.84–0.96, *p* < 0.05); this data was not included in the original study, conducted on patient with low-back pain, considered of limited utility on estimation of pain measure reliability [3].

For interrater reliability, there was a good concordance rate (83.3%) between reference diagnosis and LANSS (Cohen's kappa = 0.67, CI 95% 0.52–0.75), consistent with the original study (Cohen's kappa = 0.65) [4] and with the Korean (Cohen's kappa = 0.69) [18] and Spanish LANSS validation study (Cohen's kappa = 0.70) [8].

For the accuracy assessment, using as cutoff a value ≥12 [9], LANSS confirmed an acceptable sensitivity and lower specificity (87% and 72%, respectively) with a PPV of 83% and NPV of 79% compared to results of Bennet (sensitivity 85%, specificity 80%, PPV 81%, NPV 84%) [4]. In terms of convergent validity, all LANSS items showed a moderate to strong correlation with allodynia, which would be considered a strong association for a factor analysis.

Furthermore, PD-Q investigates peculiar details of gradation of descriptors, pain course pattern, and radiating pain. For example, for the sensory descriptors, NeP group had an intensity distribution mainly between moderate and strong, particularly for “temperature evoked pain” and “numbness sensation,” defined as absent or mostly “hardly noticed” in the nociceptive group. Instead, regarding pain course pattern, we obtained similar results in both groups. No patient referred radiating distribution in other body regions. Moreover, we reported that, in NeP group, our translated version of PD-Q was able to identify the pain course pattern specific of the neuropathic syndromes included in our study (trigeminal neuralgia and postherpetic pain) according to descriptors found in literature [10, 11].

Finally, the good concordance rate between the reference diagnosis and the mean value of LANSS confirmed that our translated version of the questionnaire may be considered a useful screening tool in daily clinical routine and in the therapeutic management of this insidious kind of pain.

As expected, using a unidimensional scale such as VAS, at the T0, we observed similar results in the two groups in terms of pain intensity (*p* > 0.05). This result suggests that a specialized approach to pain is strongly advised to discriminate between different pain phenotypes and also to consider several aspects of painful experience, particularly when chronic pain occurs.

Any disease associated to chronic pain, i.e., OA or fibromyalgia, results in temporal, spatial, and threshold modification of pain perception with consequent uncoupling between central (somatosensory system) and peripheral (nociceptors) activity, also known as central sensitization [7, 12]. This process explains how patients suffering from OA-related pain, typically described as nociceptive pain, could experience painful episodes with neuropathic features [7], and a multidisciplinary integrated approach is strongly suggested [13].

In a study that evaluated Self-Complete LANSS (S-LANSS) and PD-Q in patients with knee OA, Moreton et al. demonstrated that PD-Q is a good surrogate measure of augmented central pain processing, while it is still debated which questionnaire between S-LANSS and PD-Q is most accurate to discriminate neuropathic pain mechanisms in knee OA [19].

In addition, LANSS successfully detects neuropathic features also in rare painful conditions such as Complex Regional Pain Syndrome type I (CRPS I) [20]. This condition typically occurs after trauma or surgery, characterized by pain out of proportion compared with the severity of the inciting event [21] without a nerve injury. Moreover, CRPS I has a relevant impact in terms of disability and quality of life and often has an unpredictable clinical course [22], so an easy-to-use tool such as LANSS may be helpful in monitoring pain changes over time in this condition [23].

A limitation of our study could be that it was performed in a clinical setting, reducing generalizability of the results.

## 5. Conclusion

This study is the first validation of Italian versions of LANSS and PD-Q. Our results demonstrated good psychometric and discriminant pain features of the translated questionnaires, with appropriate patient comprehension. Moreover, our study provides more information about the test-retest reliability, not available in original validation studies.

Reliable pain assessment, including the identification of neuropathic features, is critical to plan an appropriate therapeutic strategy. This study confirms the validity of LANSS and PD-Q as screening tools for NeP symptoms, thus improving clinical judgment in the pain management.

## Figures and Tables

**Table 1:** Baseline demographic data according to pain reference diagnosis.

	NoP group (*N* = 50)	NeP group (*N* = 50)
*Gender, N (%)*		
Women	30 (60%)	35 (70%)
Men	20 (40%)	15 (30%)
Age (years–mean ± SD)	60 ± 6	50 ± 19
Weight (Kg–mean ± SD)	78 ± 15	70 ± 9
Height (cm–mean ± SD)	168 ± 8	165 ± 12
BMI (Kg/m2–mean ± SD)	28 ± 7	26 ± 3
*Education level, N (%)*		
Primary	24 (48%)	20 (40%)
High	16 (32%)	18 (36%)
University	10 (20%)	12 (24%)

Descriptive data for continuous variables are expressed as mean ± standard deviation (SD); discrete variables are expressed as numbers (*N*) and percentages (%). NoP: nociceptive pain; NeP: neuropathic pain; BMI: body mass index.

**Table 2:** VAS score, LANSS score (test/retest), and PD-Q score (test/retest) according to pain reference diagnosis.

	VAS	PD-Q score (test)	PD-Q score (retest)	*pvalue* ^*∗*^	LANSS score (test)	LANSS score (retest)	*pvalue* ^*∗*^
NeP group (*N* = 50)	6.8 ± 2.9	22.35 ± 4.8	21.5 ± 4.1	>0.05	18 ± 4.8	17.5 ± 4.6	>0.05
NoP group (*N* = 50)	6.3 ± 2.3	4.8 ± 3.7	5.2 ± 3.5	>0.05	4.4 ± 3.4	4.5 ± 3.7	>0.05
*pvalue* ^*∗*^	>0.05	<0.01	<0.01		<0.01	<0.01	

Descriptive data for continuous variables are expressed as mean ± standard deviation (SD); discrete variables are expressed as numbers (*N*);^*∗*^*pvalues* < 0.05 were considered statistically significant. NeP: Neuropathic pain; NoP: nociceptive pain; VAS: visual analogue scale; PD-Q: pain DETECT-Questionnaire; LANSS: Leeds assessment of neuropathic symptoms and Signs.

**Table 3:** Details of sections B, C, and D of PD-Q scores in NeP and NoP groups at the baseline.

Section B pain course pattern	NeP group *N* (%)	NoP group *N* (%)										
Persistent pain with slight fluctuations	9 (18%)	6 (12%)										
Persistent pain with pain attacks	16 (32%)	6 (12%)										
Pain attacks without pain between them	17 (34%)	32 (64%)										
Pain attacks with pain between them	8 (16%)	6 (12%)										
Missing responses (not answered)	0	0										
Section C does your pain irradiate to other regions of your body?	NeP group *N* (%)	NoP group *N* (%)										
Yes	8 (16%)	11 (22%)										
No	42 (84%)	39 (78%)										
Missing responses (not answered)	0	0										
Section D	NeP group *N* (%)						NoP group *N* (%)					
Pain descriptors	No	Hardly noticed	Slightly	Moderately	Strongly	Very strongly	No	Hardly noticed	Slightly	Moderately	Strongly	Very strongly
1. Do you suffer from a burning sensation (eg, stinging nettles) in the marked areas?	0	0	17 (34%)	25 (50%)	0	8 (16%)	30 (60%)	9 (18%)	11 (22%)	0	0	0
2. Do you have a tingling or prickling sensation in the area of your pain (like crawling ants or electrical tingling)?	0	0	8 (16%)	26 (52%)	8 (16%)	8 (16%)	39 (78%)	2 (4%)	9 (18%)	0	0	0
3. Is light touching (clothing, a blanket) in this area painful?	9 (18%)	0	17 (34%)	16 (32%)	8 (16%)	0	50 (100%)	0	0	0	0	0
4. Do you have sudden pain attacks in the area of your pain, like electric shocks?	0	0	9 (18%)	16 (32%)	17 (34%)	8 (16%)	33 (66%)	12 (24%)	5 (10%)	0	0	0
5. Is cold or heat (bath water) in this area occasionally painful?	0	0	0	9 (18%)	41 (82%)	0	50 (100%)	0	0	0	0	0
6. Do you suffer from a sensation of numbness in the areas that you marked?	8 (16%)	0	0	9 (18%)	33 (66%)	0	23 (46%)	17 (34%)	0	7 (14%)	3 (6%)	0
7. Does slight pressure in this area, for example, with a finger, trigger pain?	9 (18%)	0	9 (18%)	16 (32%)	8 (16%)	8 (16%)	29 (58%)	0	5 (10%)	7 (14%)	9 (18%)	0

*Note.* Discrete variables are expressed as numbers (*N*) and percentages (%). Abbreviations: PD-Q: pain DETECT- questionnaire; NeP: neuropathic pain; NoP: nociceptive pain.

## Data Availability

The data are available from Prof. Migliore upon reasonable request.
